# Recent advances in the structural molecular biology of Ets transcription factors: interactions, interfaces and inhibition

**DOI:** 10.1042/BST20130227

**Published:** 2014-01-23

**Authors:** Christopher D.O. Cooper, Joseph A. Newman, Opher Gileadi

**Affiliations:** *Structural Genomics Consortium, University of Oxford, Old Road Campus Research Building, Old Road Campus, Headington, Oxford OX3 7DQ, U.K.

**Keywords:** cancer, dimerization, Ets transcription factor, protein–DNA ternary complex, protein–protein interaction, EBS, Ets-binding site, PNT, pointed, PPI, protein–protein interaction, RHA, RNA helicase A, TF, transcription factor

## Abstract

The Ets family of eukaryotic transcription factors is based around the conserved Ets DNA-binding domain. Although their DNA-binding selectivity is biochemically and structurally well characterized, structures of homodimeric and ternary complexes point to Ets domains functioning as versatile protein-interaction modules. In the present paper, we review the progress made over the last decade to elucidate the structural mechanisms involved in modulation of DNA binding and protein partner selection during dimerization. We see that Ets domains, although conserved around a core architecture, have evolved to utilize a variety of interaction surfaces and binding mechanisms, reflecting Ets domains as dynamic interfaces for both DNA and protein interaction. Furthermore, we discuss recent advances in drug development for inhibition of Ets factors, and the roles structural biology can play in their future.

## Introduction

**Figure 1:**
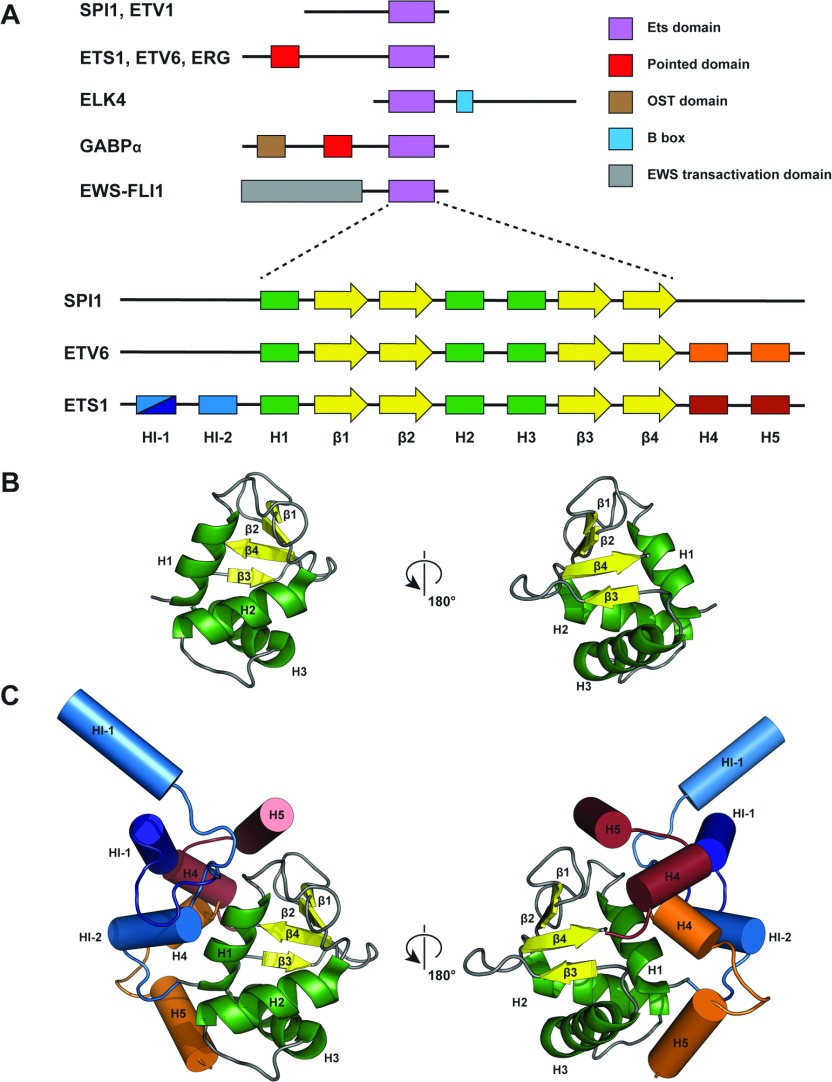
Structure of Ets domain transcription factors (**A**) Domain architecture of representative members of the Ets family, with domains and lengths not to scale. Only structured domains rather than transactivation/inhibitory domains are shown (upper panel). Expansion represents the core Ets domain secondary structure (lower panel). Core Ets domain secondary-structural elements are green rectangles (α-helices/H) and yellow arrows (β-sheet/β), with appended α-helices shown where appropriate. (**B**) Cartoon representation of a typical core Ets domain fold (ELK4, PDB code 1K6O [11]). Secondary-structural elements are coloured and labelled according to (**A**). (**C**) Structural diversity amidst a conserved core. Cartoon representation of a core Ets fold (ELK4), with superimposed appended helices represented as cylinders where appropriate. Secondary-structural elements are coloured and labelled according to (**A**). Two alternative conformations of ETS1 helix HI-1 are shown (light blue, uninhibited, PDB code 3MFK [15]) and autoinhibited (dark blue, PDB code 1R36 [36]), with the inhibited ETV6 additional helices represented in orange (PDB code 2DAO [38]).

The Ets TF (transcription factor) family is found throughout the metazoa, comprising 28 members in humans [1,2], all containing the evolutionarily conserved DNA-binding Ets domain which binds the invariant DNA sequence 5′-GGA(A/T)-3′ [2]. Ets TFs play important roles in normal cellular development and differentiation [3], but when deregulated are significant mediators of tumorigenesis in various cancers [4,5]. Ets proteins are subclassified by the presence of further domains associated with PPIs (protein–protein interactions) or transcriptional regulation [2,3], including the TCF (containing the B-box [6]) and PEA3 (containing the unstructured PEA3 transactivation domain [7]) subfamilies. PNT (pointed) domains are also frequently found N-terminal to the Ets domain, involved in PPI and homodimerization [8,9] (Figure 1A).

Although different Ets TFs may bind similar DNA sequences and are expressed in multiple cell-dependent combinations, much of their binding specificity and regulation is mediated directly by the structurally conserved Ets domain. A number of Ets protein structures have been determined, either individually or as binary or ternary complexes with interaction partners or DNA (comprehensively listed elsewhere [2] and in Table 1). These structures have shed light on some mechanisms used to regulate Ets function, particularly interaction interfaces utilized in binding co-operativity and sequence selectivity of Ets ternary complexes on tandem DNA motifs [10–12]. A number of homodimeric Ets domain structures have also helped to elucidate mechanisms of autoinhibition of DNA binding compared with co-operative binding [13–15].

**Table 1:** Ets domain complexes and structural information The list is not exhaustive, but provides an overview of the variety of Ets protein interactions characterized biochemically and structurally in the literature. N/R, no record.

Ets domain	Interaction type	Protein partner	PDB code	Interface details	Reference
ETS1	Homodimer	ETS1	2NNY	Head-to-head Ets domains on palindromic EBS. HI-2 and HI-2/H1 loop contact H2-H3 loop reciprocally (‘Area I’)	[42]
ETS1	Homodimer	ETS1	3MFK	Similar to Area I in 2NNY on palindromic EBS. Additional contacts between dimer units, with HI-1 contacting H4, HI-2 and HI-1/HI-2 loop reciprocally (‘Area II’)	[14]
ETS1	Homodimer	ETS1	3RI4	Interface similar to Area II from 3MFK, but on two separate EBS units	[15]
ETS1	Heterodimer	PAX5	1K78	PAX5 β-hairpin Gln^22^ hydrogen-bonds to reposition ETS1 Tyr^395^ in Ets helix H3	[12]
ETS1	Heterodimer	FOXO1	4LG0*	Ets domain interaction	N/R
ETS1	Heterodimer	AML1	3W46†	Autoinhibitory regions from each partner interact to reciprocally relieve inhibition of DNA binding	[64]
ELK4 (SAP1)	Heterodimer	SRF	1K6O	SRF MADS domains N-terminal Leu^155^ fits into small pocket comprising Ets H1, H4 and H5 and the H3 on Ets H3, reorienting Tyr^65^ and Arg^64^ to enhance DNA binding	[11]
SPI1 (PU.1)	Heterodimer	IRF4	N/R	Ets H2-H3 loop contacts IRF4 across the DNA minor groove	[48]
SPI1 (PU.1)	Heterodimer	AP-1 (JUN)	N/R	Jun basic domain binds Ets β3-β4 region	[65]
SPI1 (PU.1)	Heterodimer	NF-IL6	N/R	Ets domain interacts through β2-α2-α3 and β3-β4 elements	[53]
GABPα	Heterodimer	GABPβ	1AWC	GABPβ ankyrin repeat loops bind cleft comprising Ets H1, H4 and H5 and the H3-β3 loop	[47]
ERG	Heterodimer	DNA-PKcs	N/R	Involves Tyr^373^ at the edge of the H3 helix	[29]
ERG	Heterodimer	AR	N/R	Involves Ets domain H3-β3-β4 region	[54]
ERG	Heterodimer	AP-1 (JUN)	N/R	Jun basic domain binds Ets H3 region	[52]
ETV1	Heterodimer	AR	N/R	Involves Ets and upstream regions	[66]
ETV1	Homodimer	ETV1	4AVP, 4BNC	Reciprocal contacts between H1, H4 and the β1-β2 loop; significant hydrophobic area and intersubunit disulfide bond	(C.D.O. Cooper, J.A. Newman, C.K. Allerston and O. Gileadi, unpublished work)
FEV	Homodimer	FEV	2YPR, 3ZP5	Reciprocal contacts between H1, H4 and the β1-β2 loop; significant hydrophobic area and intersubunit disulfide bond	(C.D.O. Cooper, J.A. Newman, C.K. Allerston and O. Gileadi, unpublished work)
ELK1	Homodimer	ELK1	1DUX	Reciprocal contacts between Ets H1/H1-β1 loop	[43]

*PDB record on hold

^†^One of many PDB records on hold

Such structural studies have demonstrated the evolution of multiple independent PPI interfaces on Ets domains, thereby illustrating their versatile nature, not only responsible for binding DNA, but also critical for the regulation of DNA binding and transcriptional activity. As Ets proteins are central to cancer development and progression, Ets PPI interfaces are clear targets for abrogation by chemotherapeutic drugs [5,16]. In the present paper, we review existing and recent progress on structural studies of Ets interaction and interfaces, with the prospect of exploiting these surfaces as drug targets and to further our understanding of Ets regulation.

## Ets proteins in biology and cancer development

Ets TFs are expressed ubiquitously or in tissue-specific patterns [17] and are particularly involved in differentiation processes such angiogenesis [18] and haemopoiesis [19]. PEA3 proteins play particular roles in branching morphogenesis and limb development [20]. Genetic knockouts suggest functional redundancy of some Ets factors [21], and genome-wide analyses show both specific and redundant Ets occupancy in promoter-proximal regions [22], reflecting plasticity in Ets transcriptional regulation.

As Ets TFs regulate activation or repression of key developmental or homoeostatic target genes, it is not surprising that Ets deregulation is a driving force in neoplastic transformation, metastasis and progression [4]. Ets overexpression may follow chromosome rearrangements, from copy gains of *ETV1* in melanoma [23], to fusion of *ERG* or *ETV1* to the *TMPRSS2* promoter, resulting in androgen-inducible expression in prostate cancer [24], associated with aggressive disease [25]. Chromosomal translocations are prevalent in Ewing's sarcomas, where the EWS transactivation domain is fused to Ets domains of ETV1, ETV4, ERG, FLI1 or FEV, dominantly activating transcription of Ets targets [26]. Cancer development is hence likely to be mediated by Ets target genes driving various stages of the neoplastic process, e.g. immortalization following hTERT (human telomerase reverse transcriptase) up-regulation [27] or E2F cell cycle disruption [28], increased DNA damage [29], or metastasis following matrix metalloproteinase up-regulation [30].

## Ets transcription factor structure

Ets TFs are modular proteins with the Ets domain generally present at either terminus [2] (Figure 1A). Ets domains comprise a small (~85-residue) four-stranded antiparallel β-sheet packed against three semi-orthogonal α-helices in a variant helix–turn–helix (winged helix) conformation [31] (Figures 1A and 1B). Ets domains can bind ~15 bp dsDNA with a 10 bp specificity at EBSs (Ets-binding sites), where the H3 helix acts in DNA recognition by inserting in the major groove, allowing conserved arginine and tyrosine residues to hydrogen-bond bases in the consensus 5′-GGA(A/T)-3′ motif [2]. Ets proteins are grouped into four classes on the basis of DNA-binding specificity, reflecting residues in helix H3 and the H3–β3 loop [32]. The mechanism for DNA sequence recognition outside the GGA(A/T) core is less clear, with indirect readout suggested as a contributing factor [33]. Given this overlap in Ets recognition sequences, further specificity is extended by combinatorial and co-operative binding with other TFs [10] at tandem (e.g. ETS1/RUNX [34]) or palindromic sites (ETS1) [35] respectively.

DNA binding may be regulated by sequences bordering the Ets; for instance, ETS1 DNA binding is inhibited by two helices flanking each side of the Ets. These form a helical bundle which packs against helix H1 distal to the DNA-binding face [13] (Figure 1C), with the metastable HI-1 of the inhibitory bundle unfolding on DNA binding [36]. Studies on ERG suggest allosteric inhibition may result from stabilization of the conformation of a conserved tyrosine residue on helix H3, which is less optimal for DNA binding, or by reducing polypeptide backbone dynamics in the inhibited state [37]. In a further mechanism, two helices appended to the ETV6 Ets C-terminus can inhibit DNA binding by steric blocking [38] (Figure 1C).

## Ets domains as protein–protein interaction modules

Many eukaryotic TFs act as non-covalent dimers, with interaction critical for function, mediated by DNA-binding domains or through additional subunits [39]. Ets TFs can dimerize using the Ets domain and/or additional domains such as PNT [9], with Ets-mediated interactions either homodimeric or heterodimeric with other TFs or protein partners (Table 1). Homodimerization allows co-operative binding to repeated DNA elements [35], with heterodimeric interactions with non-Ets proteins potentiating combinatorial control of DNA binding [40], crucial for tissue-specific transcriptional regulation.

### Homodimeric Ets complexes

**Figure 2:**
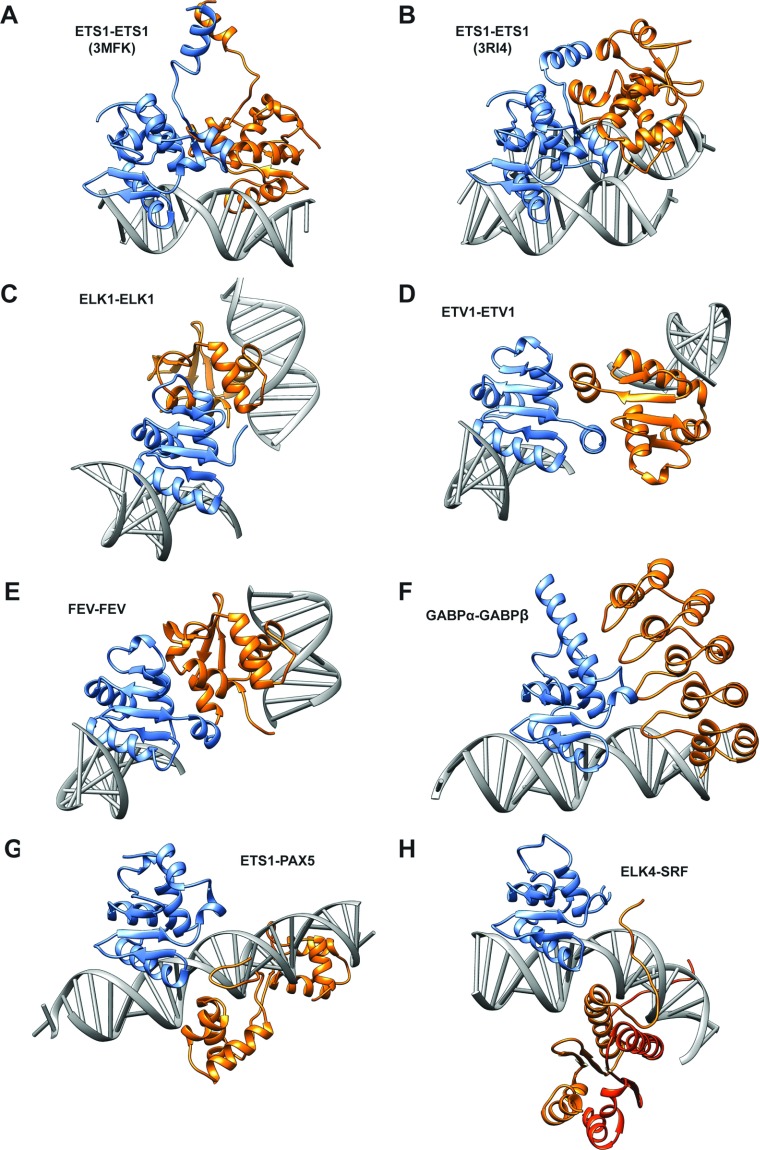
Structural comparison of Ets homo- and hetero-dimeric complexes Ets ternary PDB structures were superimposed against one Ets subunit (blue), whereas the relative position of interacting Ets or other protein partners are in orange. Additional subunits not interacting with Ets domains are in red/orange, with dsDNA in grey. (**A**) ETS1 (PDB code 3MFK) showing Area I interface (note that HI-1 helices do not contact). (**B**) ETS1 (PDB code 3RI4) showing Area II interface. (**C**) ELK1 (PDB code 1DUX). (**D**) ETV1 (PDB codes 4AVP and 4BNC). (**E**) FEV (PDB codes 2YPR and 3ZP5). (**F**) GABPα–GABPβ (PDB code 1AWC). (**G**) ETS1–PAX5 (PDB code 1K78). (**H**) ELK4–SRF (PDB code 1K6O).

Perhaps the most structurally studied Ets protein is ETS1 [41], existing as an autoinhibited monomer in solution, although domain-swapped dimers have been crystallized in the absence of DNA [13]. Monomeric ETS1 can bind to single EBS motifs, or co-operatively in dimeric configurations at palindromic sites such as the stromelysin-1 promoter [35], thereby counteracting its autoinhibition. Two protein interface areas are observed in different ETS1–DNA ternary structures, with Area I involving a head-to-head dimeric arrangement orthogonal to the DNA-binding face (PDB codes 2NNY [42] and 3MFK [14]) (Figure 2A), and Area II involving domain-swapped interactions between two sets of juxtaposed ETS1 dimer units (3MFK [14] and 3RI4 [15]). Area I comprises reciprocal hydrogen bonds and van der Waals interactions from helix HI-2 and the HI-2/H1 loop to the H2-H3 loop, between opposing subunits. This buries ~370 Å^2^ (1 Å=0.1 nm) of monomer surface and the 4 bp spacing between palindromic EBSs is critical for this interaction as the HI-2/H1 loop interacts with the minor groove in this region. The Area II domain-swapped interface between two sets of dimers buries 650 Å^2^, with the N-terminus of HI-1 contacting H4, HI-2 and the HI-1/HI-2 loop on the opposing dimer. Recently, a similar Area II interface was reported for another dimeric ETS1 configuration, with an ETS1 dimer complexed to two separate dsDNA duplexes in an antiparallel EBS configuration (PDB code 3RI4 [15]) (Figure 2B). Here, additional hydrogen bonds are found between one ETS1 subunit and neighbouring DNA bound by the other ETS1 subunit. Although the ETS1 (PDB code 3RI4) Area II interface and local structure is similar to ETS1 (PDB code 3MFK), the Ets domains are in differing orientations allowing the DNA to run parallel, reflecting flexibility of the N-terminal region. This arrangement may allow widely separated EBSs to be brought together by looping *in vivo*, potentially at nucleosomes [15].

Homodimeric arrangements are found in further Ets domains; for instance, the TCF member ELK1 crystal structure (1DUX [43]) (Figure 2C) has a reciprocal interface between monomers at the C-terminal end of the helix H1 and H1-β1 loop, involving three pairwise hydrogen bonds [44]. Although this buries a similar area to ETS1 (480 Å^2^), the dimerization interface is different as ELK1 does not have the additional helices of ETS1. This arrangement orients the ELK1 monomers with their DNA-binding faces on nearly opposite sides of the dimer interface, hence they could not bind closely separated EBS motifs, unlike the head-to-head ETS1 structure. ELK1 Ets dimerization is observed *in vitro* and *in vivo* with the H1-β1 loop required for dimerization and cytoplasmic stability [45]. Recent structures of ETV1 (PDB code 4AVP) (Figure 2D) and FEV (PDB code 2YPR (Figure 2E) Ets domains reveal a further significant dimeric interface (C.D.O. Cooper, J.A. Newman, C.K. Allerston and O. Gileadi, unpublished work), with reciprocal contacts involving H1, H4 and the β1-β2 loop burying >700 Å^2^. Although contacts are predominantly non-polar, a hydrogen bond and significantly, an intermolecular disulfide linkage are also present. This interface juxtaposes ETV1 subunits in a different orientation and surface position relative to FEV and the ELK1 homodimers, although similarly positioning the H3 helix to preclude binding close or tandem EBS sequences.

### Complexes of Ets and other proteins

Ets factors are promiscuous partners in PPI networks [9,40], with a number of structurally characterized heterodimeric ternary interactions on DNA involving Ets domains, elucidating the role of interaction interfaces [10]. The GABP TF heterodimeric structure (PDB code 1AWC) (Figure 2F) comprises the Ets domain of the GABPα subunit complexed with the ankyrin repeat-containing GABPβ subunit [47]. The interface buries a significant 1600 Å^2^ in total, mainly involving hydrophobic contacts, but also some water-mediated hydrogen bonds. This involves the tips of the GABPβ ankyrin repeats fitting into a depression formed by H1, H4 and H5 of GABPα, along with the H3-β3 loop. Although this positions GABPβ in a similar relative juxtaposition to one subunit in the ETV1 dimer, this interface is specific for GABPα as ETS1 cannot bind GABPβ [47]. Whereas the GABPα/β heterodimer binds DNA with greater affinity than GABPα alone, GABPβ does not contact DNA directly. Instead, an indirect hydrogen bond from GABPβ Lys^69^ to GABPα Gln^321^ to the DNA phosphate may strengthen interaction, but reorientation of the H5 helix away from the DNA interface could also be involved, analogous to ETS1 autoinhibition [47].

A very different Ets domain ternary interface is illustrated by the ETS1–PAX5 complex with the *mb-1* promoter (PDB code 1K78) [12]. Here, one of the PAX5 paired domains binds its cognate DNA on the opposite side of the DNA duplex from ETS1, with only 180 Å^2^ of monomer surface buried in the ETS1–PAX5 interface. Yet, this interaction is critical for binding the low-affinity *mb-1* promoter, as the PAX5 β-hairpin Gln^22^ repositions the conserved Tyr^395^ side chain in ETS1 helix H3 to form more optimal DNA contacts. In addition, PAX5 forms further van der Waals and salt bridge contacts to ETS1. Hence the DNA-binding H3 helix is key to both protein–DNA and PPIs. This is analogous to the heterodimeric interface from the SRF–ELK4 (SAP1) ternary structure with DNA (PDB code 1K6O [11]). Although the primary interaction of the SRF MADS domain is with the ELK4 C-terminal B box, a similar stabilization of optimal DNA contacts as seen with ETS1–PAX5 occurs following SRF binding to its cognate DNA sequence and ELK4. Here, a small hydrophobic pocket comprising residues from the ELK4 helix H3 accommodates the N-terminal SRF Leu^155^, reorienting a conserved tyrosine and arginine residue to make additional DNA contacts [11]. SPI1 (PU.1) also forms an interface across the minor groove with IRF4, where IRF4 binding increases co-operativity up to 40-fold, presumably involving the shift of SPI1 from participating in a salt bridge with the DNA backbone to one with IRF4 [48]. Hence binding of Ets domains to their heterodimeric partners allows a dynamic change in co-operative binding properties, with SPI1 gaining binding energy from interacting with IRF4, and both PAX5 and SRF assisting ETS1, not by providing binding energy, but from optimizing binding to low-affinity DNA sequences.

Although further Ets heterodimeric interactions have been reported, structural information is currently scarce (Table 1). The heterodimeric AP-1 TF is involved in cell proliferation [49] and binds to a number of Ets domains through the Jun basic domain [50]. SPI1 interacts via the β3/β4 elements close to helix H3 where GATA TFs compete to bind and hence repress SPI1 transactivation [51]. The β3/β4 region is not required for interaction with ERG, however [52], reflecting functional diversity within this structural scaffold. Instead, the ERG helix H3 Tyr^371^ and Arg^367^ are critical for Jun interaction [52]. Of particular note, the proximal Tyr^373^ at the edge of ERG helix H3 can mediate an interaction with DNA-PKcs (DNA-dependent protein kinase catalytic subunit) directly in a DNA-independent manner, required for ERG-driven transcription and neoplastic effects [29]. SPI1 also interacts with NF-IL6 (nuclear factor for interleukin 6 expression) [C/EBPβ (CCAAT/enhancer-binding protein β)] through the β2-α2-α3 and β3-β4 regions [53]. The importance of these additional Ets interaction partners merits structural study, as some interfaces appear to be novel and even if such surfaces appear similar to those already characterized, subtle residue movements are key to co-operative binding on DNA [11,12]. Furthermore, many structurally uncharacterized Ets domain interactions involve other TFs central to cellular development or neoplasia, such as the androgen receptor [54], HOX homeodomains [55] and forkhead TFs, although an ETS1–FOXO1 (forkhead box O1) structure (PDB code 4LG0) is currently on hold in the PDB.

### Ets domains as conserved yet versatile interaction interfaces

Although Ets domains act as flexible PPI modules with multiple partners as described [9,40] (Table 1), they exhibit strong sequence and structural identity. Interaction specificity is therefore likely to be determined by appended helices and small residue substitutions (e.g. SPI1 lacks two conserved tyrosine residues in helix H3). Relative juxtapositioning of Ets interaction partners demonstrates that structurally determined interaction interfaces are limited to two areas (Figure 2). Interactions involving the DNA-recognition helix H3 across the DNA duplex unsurprisingly associate with structural changes relating to DNA-binding modulation. Other interfaces are on the face containing helix H1 and appended helices, but bound at a variety of sites within this region. This may reflect the diversity of appended helices providing multiple binding solutions (Figure 1C). Of note, however, is the lack of involvement of the opposite face containing the β3-β4 loop and surface of the β-sheet (Figure 2). As described, a number of biochemically characterized interactions involve these regions (e.g. SPI1–AP-1 [51]), hence future structural determination of such interactions is important.

## Ets transcription factors as targets for inhibition in cancer

TFs play a direct role in transformation and metastasis during cancer development, and their modulation or inhibition has long been a major aim of translational cancer research [56]. As described above, overexpressed Ets TFs are major players in cancer development and can drive aggressive disease, hence they have been identified as significant targets for drug development [5,56]. A number of strategies are available for TF drug targeting [16], and early attempts to target Ets TF–DNA interactions used oligonucleotides to mimic the EBSs and saturate ETS1 [57], or target EBS motifs directly to bind the minor groove in a sequence-dependent manner, occluding Ets domain binding [58]. The similarity of EBS motifs, however, could limit this method, but abrogation of Ets PPI interfaces presents an attractive alternative avenue for small-molecule targeting. These have promise as potential inhibitors, but, although PPI interfaces often display a lack of defined binding pockets, small molecules often have pharmacokinetic properties superior to those of peptide inhibitors [16] and greater chemical space can be explored [59]. For instance, the imidazoline derivative Nutlin-3a inhibits the p53–MDM2 (murine double minute 2) interaction, potentiating p53-dependent cell cycle arrest [60]. Ets domains are promising PPI inhibition targets, as structural analysis illustrates multiple yet specific interaction surfaces and potentially druggable pockets, e.g. a small hydrophobic pocket in ELK4 accommodating the SRF Leu^155^ side chain [11,12], and a cleft in GABPα binding the GABPβ ankyrin repeat loops [47]. Although some Ets interfaces are small, disruption may be adequate to abrogate conformational movements that otherwise occur on binding e.g. ETS1–PAX5 [11,12].

A significant development has been the isolation of YK-4-279, an inhibitor of the oncogenic EWS–FLI1 Ets fusion in Ewing's sarcoma identified by library screening to inhibit binding to its interaction partner RHA (RNA helicase A) [61]. Although YK-4-279 binds EWS–FLI1 weakly (*K*_d_ ~10 μM), it inhibits EWS–FLI1 in a dose-dependent manner *in vivo* and in xenografts [61], with (S)-YK-4-279 determined as the active enantiomer [62]. As EWS–FLI1 includes the FLI1 Ets portion and some upstream regions, both RHA and YK-4-279 potentially directly bind the Ets domain, particularly as YK-4-279 also inhibits ERG and ETV1-mediated invasion in prostate cancer [63]. YK-4-279, however, is likely to inhibit PPIs other than RHA with ERG–ETV1, suggesting a potentially different binding site for YK-4-279 [63]. Structural studies of the interaction of YK-4-279 would augment current research, not only to identify the binding site on EWS–FLI1 and ERG–ETV1, but also to assist in structure-based drug design, increasing the affinity of YK-4-279 for its targets to potentially increase its potency and specificity for the Ets TF with which it interacts.

## Concluding remarks

The structural biology of Ets DNA binding is advanced, with nuances of DNA-binding selectivity and autoinhibition well studied [2]. The variety of Ets domain structures demonstrate that, although Ets core architectures are similar and comprise highly conserved sequences, they are versatile and encompass multiple dynamic PPI surfaces, involving a variety of bonding types. Although a number of heterodimeric Ets structures have illustrated the role of interfaces in modulating DNA binding, most Ets complex structures in the last decade have been of homodimers. Although this has increased understanding of the mechanism of autoinhibitory control, a large number of structurally unresolved Ets interactions remain. Structural analyses of such protein partnerships are essential to elucidate the role of further interfaces such as the Ets β-sheet face, particularly as many of these partnerships play key roles in cancer. Hence further structural studies are essential to assist drug targeting of Ets TFs in cancer, to increase the scope of Ets surfaces available for inhibition and to increase the potency of existing drugs. Although Ets targeting has taken a huge step forward with the development of the small-molecule inhibitor YK-4-279, future structural analysis can assist drug development, from resolving mechanisms of inhibitor binding, to increasing drug specificity and potency. Thus, as interactions, interfaces and inhibition are closely linked, for any vote on the importance of structural biology in Ets research, the ‘I's certainly have it.
